# A short guide to long non-coding RNA gene nomenclature

**DOI:** 10.1186/1479-7364-8-7

**Published:** 2014-04-09

**Authors:** Mathew W Wright

**Affiliations:** 1 HUGO Gene Nomenclature Committee (HGNC), EMBL-EBI, Wellcome Trust Genome Campus, Hinxton, Cambridge, CB10 1SD, UK

**Keywords:** Long non-coding RNA, Nomenclature, ncRNA, lncRNA

## Abstract

The HUGO Gene Nomenclature Committee (HGNC) is the only organisation authorised to assign standardised nomenclature to human genes. Of the 38,000 approved gene symbols in our database (http://www.genenames.org), the majority represent protein-coding (pc) genes; however, we also name pseudogenes, phenotypic loci, some genomic features, and to date have named more than 8,500 human non-protein coding RNA (ncRNA) genes and ncRNA pseudogenes. We have already established unique names for most of the small ncRNA genes by working with experts for each class. Small ncRNAs can be defined into their respective classes by their shared homology and common function. In contrast, long non-coding RNA (lncRNA) genes represent a disparate set of loci related only by their size, more than 200 bases in length, share no conserved sequence homology, and have variable functions. As with pc genes, wherever possible, lncRNAs are named based on the known function of their product; a short guide is presented herein to help authors when developing novel gene symbols for lncRNAs with characterised function. Researchers must contact the HGNC with their suggestions prior to publication, to check whether the proposed gene symbol can be approved. Although thousands of lncRNAs have been predicted in the human genome, for the vast majority their function remains unresolved. lncRNA genes with no known function are named based on their genomic context. Working with lncRNA researchers, the HGNC aims to provide unique and, wherever possible, meaningful gene symbols to all lncRNA genes.

## Introduction

Since its inception in the 1970s, the HUGO Gene Nomenclature Committee (HGNC) [1] has kept apace with the discovery and characterisation of new human genes, providing each gene with a unique symbol and name and thus aiding effective scientific communication. By the time the initial sequence of the Human Genome was published in 2001 [2], the HGNC database (http://www.genenames.org) [3] contained more than 13,000 approved gene names, mostly for protein-coding genes with only around 200 non-coding RNA (ncRNA) gene names. With the burgeoning research and interest in ncRNAs over the last decade, the number of ncRNA loci with gene names has vastly expanded to more than 8,500 currently; about 2,000 of these represent long non-coding RNA (lncRNA) genes. Whereas classes of small ncRNAs can be defined by their shared homology and common function [4], in contrast, lncRNA genes are a disparate set of loci related only by their size (more than 200 bases in length), are non-homologous, and have variable functions [5]. Their discovery has been further complicated because they are expressed at very low levels, sometimes only at specific developmental stages, and in specific tissues [6]. Large-scale transcriptomic analyses, such as RNA-Seq, have now revealed thousands of putative long non-coding RNAs [7]; these present unique nomenclature challenges, especially because for the vast majority, the function of the resultant transcript(s) remains unknown. Below, we present a brief guide to the nomenclature of lncRNA genes and provide examples of some of the genes named to date.

## lncRNA gene naming guidelines

The HGNC endeavours to approve symbols and names that have been used in publications, but this is not always possible. To ensure their symbol can be approved authors must contact the HGNC prior to publication to agree the nomenclature for each novel lncRNA gene. When creating a new lncRNA gene name, there are a number of factors that should be taken into account:

### Each approved gene symbol must be unique

This is the paramount nomenclature rule and cannot be broken. Uniqueness enables unambiguous communication and this utility of approved gene nomenclature ensures that everyone knows they are speaking about the same gene. If an author publishes a lncRNA name that is already in use for another locus, then the HGNC will have to assign an alternative symbol. For instance, a novel lncRNA required to keep epidermal cells in an undifferentiated state was published as *ANCR*[8] but this could not be approved since this was already in use for the ‘Angelman syndrome chromosome region’; so, in agreement with the authors, it was approved as *DANCR* for ‘differentiation antagonizing non-protein coding RNA’.

### Symbols are short-form representations of the descriptive gene name

Each lncRNA is assigned a gene symbol that is an abbreviation or acronym of a descriptive name. For example, the symbol *BANCR* is an abbreviation of the full name ‘BRAF-activated non-protein coding RNA’. Gene symbols are the primary descriptors used in communications about genes and their brevity makes them user friendly.

### Symbols should only contain Latin letters and Arabic numerals

Gene symbols should only contain Latin letters and Arabic numerals, e.g. *NEAT1* (nuclear paraspeckle assembly transcript 1). Punctuation is not used and will generally be removed or replaced by a letter or number. The use of hyphens is limited to specific exceptions, such as genes named as antisense to protein-coding genes (discussed later), e.g. *BACE1*-*AS* (BACE1 antisense RNA).

### Human gene symbols are all uppercase

By long-established convention, all human gene symbols are written in uppercase letters. This distinguishes them from rodent genes where only the first letter is uppercase and the rest lowercase. For instance the mouse gene *Hotair* is the ortholog of the human *HOTAIR* (HOX transcript antisense RNA) gene.

### Symbols should not contain any reference to species

Symbols should not contain any reference to species, for example ‘H/h’ for human. The use of ‘human’ in gene names should also be avoided because approved human gene names are transferred across to homologous genes in other species, where ‘human’ would be potentially confusing and misleading.

### Symbols should not spell out commonly used words

Whilst authors might be tempted to use commonly used words for gene symbols because they are easily recognized and pronounced, they should be avoided because they generate unnecessary confusion and make searching for information about a gene much more difficult. A good example of this is *AIRN*, which was first published as AIR [9]. A search with ‘AIR’ in PubMed returns more than 220,000 unrelated hits, whereas a search with the approved symbol ‘*AIRN*’ returns only the 10 publications specific to this gene. Other examples include EGO [10], since approved as *EGOT* (eosinophil granule ontogeny transcript), and PANDA [11] now *PANDAR* (promoter of CDKN1A antisense DNA damage activated RNA).

### If possible, names should be based on function

Genes are preferentially named based on the function of the gene product. Examples include the well-known '*XIST*' which is short for 'X (inactive)-specific transcript' because the transcript is involved in transcriptionally silencing one of the pair of X chromosomes, and more recently ‘*TINCR*’ [12] which stands for ‘tissue differentiation-inducing non-protein coding RNA’ because the product is required for epidermal tissue differentiation. If possible, the name of a gene should be based on the normal function of the gene product and not a mutant phenotype. Gene names should be concise and not attempt to represent all known information about a gene. The following are a few other things to consider in gene symbols and names:

•Must not be offensive or pejorative

•Must not be used to acknowledge individuals or places

•Should not reference names of mythical, fictional, or historical figures

•Should not be whimsical or impart no meaningful information about the gene

### Functional transcribed pseudogenes should retain their pseudogene name

A small number of transcribed pseudogenes have now been shown to be functional, e.g. *PTENP1* regulates levels of *PTEN* by binding to *PTEN*-targeting miRNA [13]. Transcribed pseudogenes with published function will retain their pseudogene nomenclature and not be renamed based on function; however, ‘(functional)’ is added to the end of the gene name so that these genes can be found in a search, e.g. the full name of *PTENP1* is ‘phosphatase and tensin homolog pseudogene 1 (functional)’.

### Naming genes with no known function

**Figure 1:**
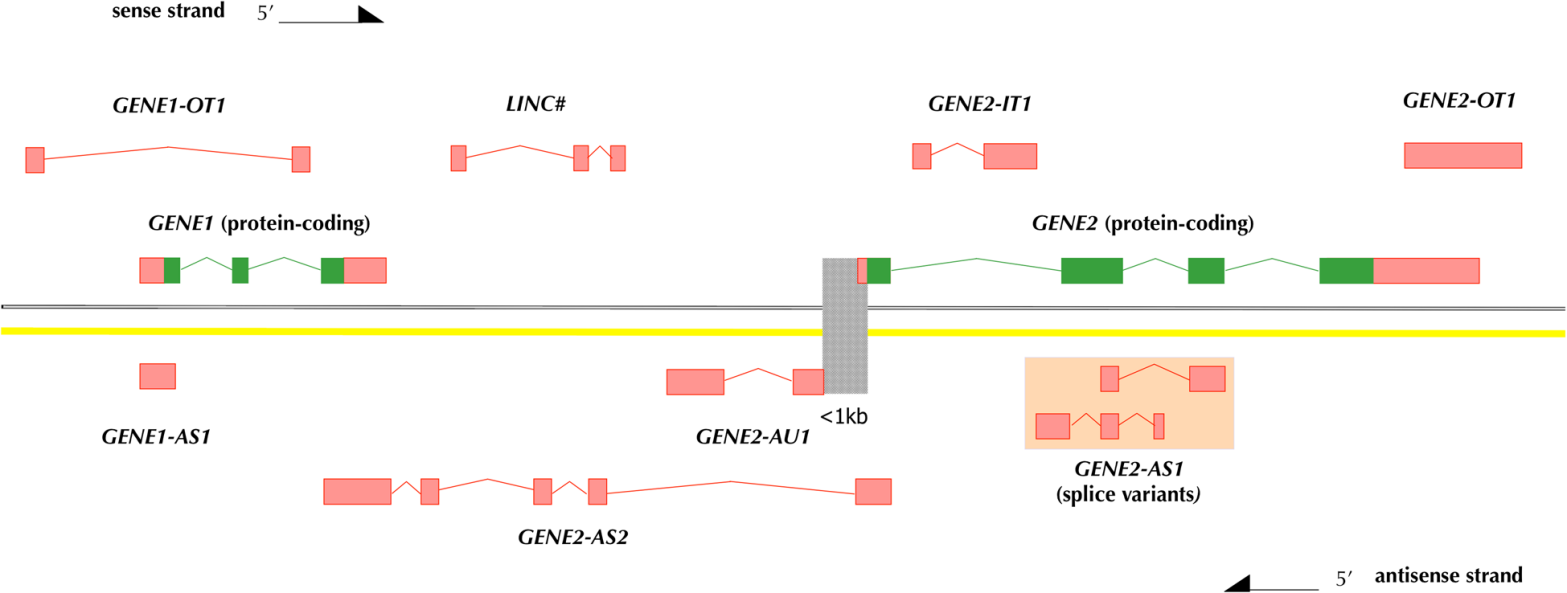
A schematic summary of the nomenclature scheme for human long ncRNA genes of no known function.

LncRNA genes with no known function are named pragmatically based on their genomic context. A schematic of the naming protocol is presented in Figure 1. This figure demonstrates how gene nomenclature can be applied in these instances but should not be used independently by researchers to generate lncRNA gene names with potentially different numbering to the approved HGNC names. If there is a proximal pc gene then the lncRNA genes are given a gene symbol beginning with the pc symbol and assigned a suffix according to whether they are: antisense (AS) e.g. *BACE1*-*AS*; intronic (IT) e.g. *SPRY4*-*IT1*; or overlapping (OT) e.g. *SOX2*-*OT*. Long intergenic lncRNAs (lincRNAs) that lie between pc gene loci are named with a common root symbol (*LINC*, ‘long intergenic non-coding RNA’) and an iterated, numerical suffix. The HGNC naming schema is consistent with the lncRNA categories annotated by GENCODE: antisense RNAs, sense intronic, sense overlapping, and lincRNA [14]. A new locus category is under consideration for lncRNAs that lie in a head-to-head orientation with a pc gene and hence putatively share a bidirectional promoter; the HGNC proposes naming these as antisense upstream (AU), e.g. *GENE2*-*AU1*. It should be noted that the HGNC does not approve names for splice variants so the two variant transcripts opposite *GENE2* in Figure 1 are named as one lncRNA gene (*GENE2*-*AS1*). Also if an lncRNA gene encodes transcripts that span more than one protein-coding gene, then the first protein-coding gene from the 5′ end of the lncRNA is used to name it, e.g. *GENE2*-*AS2* in Figure 1. This naming schema is applicable to most lncRNA genes but some lncRNA genes within gene dense regions may not fit into these discrete categories and require individual assessment by the HGNC (Additional file 1: Figure S1 shows the HGNC decision tree for naming lncRNAs with no known function).

## Conclusions

Working together with the lncRNA community, the HGNC aims to provide informative names for all lncRNA genes in the human genome. The simple guidelines stated in this paper are intended to guide researchers, but the only way to approve a new lncRNA gene symbol is to contact the HGNC. For further information on lncRNA nomenclature please see the HGNC lncRNA webpage: *http://www.genenames.org/rna/LNCRNA* and email us at hgnc@genenames.org.

## Competing interests

The authors declare that they have no competing interests.

## Supplementary Material

Additional file 1: Figure S1 HGNC decision tree for naming lncRNAs with unknown function. Click here for file
