# Supplementary figures and images for: A short guide to long non-coding RNA gene nomenclature
Source: Hum Genomics. 2014 Apr 9;8(1):7. doi: 10.1186/1479-7364-8-7 (PMC4021045; doi:10.1186/1479-7364-8-7)

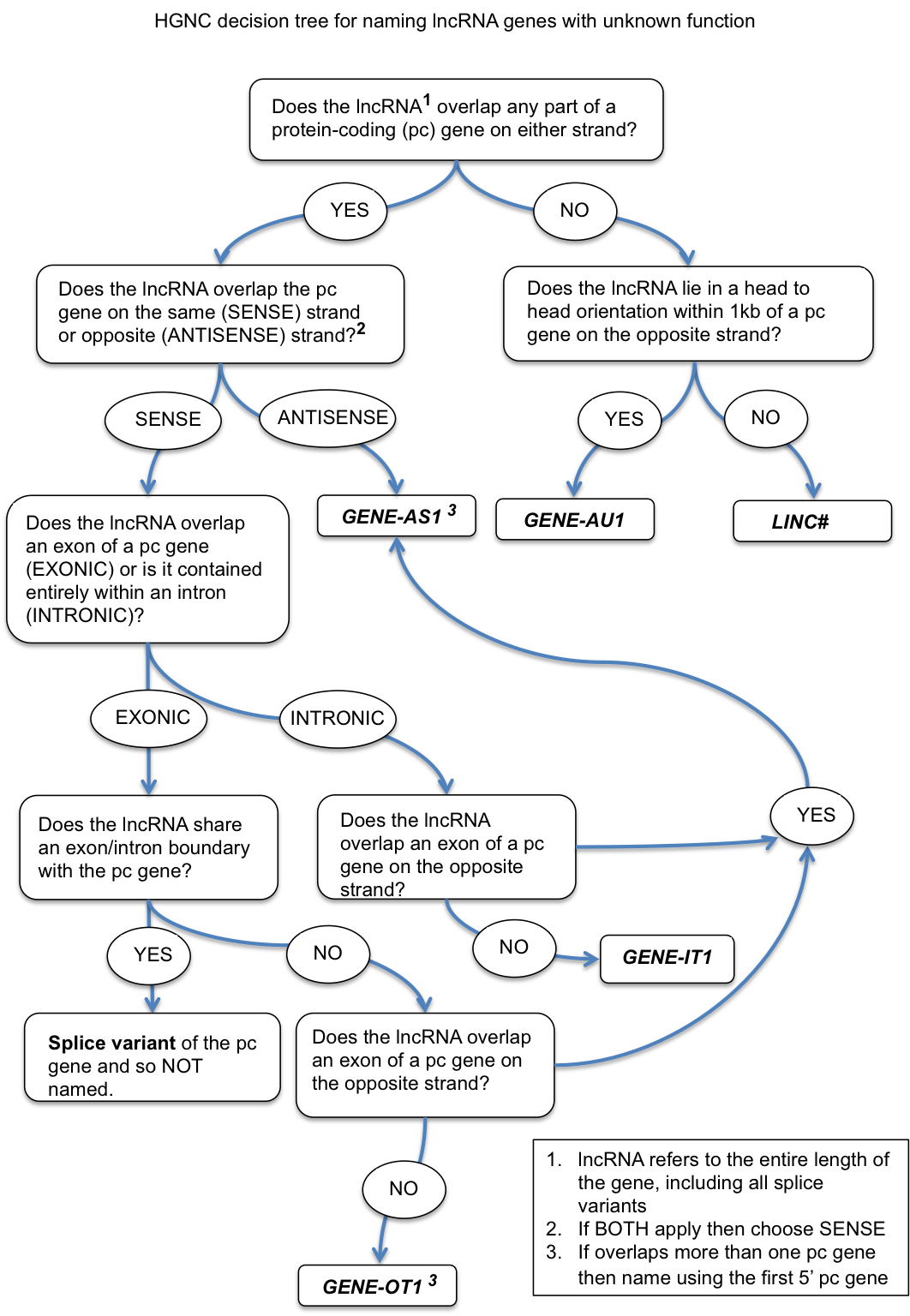

Supplement: Additional file 1: Figure S1 — HGNC decision tree for naming lncRNAs with unknown function. [file 1479-7364-8-7-S1.png]
